# The Great Meeting of Voluntary Hospital Managers

**Published:** 1911-12-02

**Authors:** 


### THE GREAT MEETING OF VOLUNTARY HOSPITAL MANAGERS.

### First List of Hospitals Represented.

Addenbrooke's Hospital, Cambridge; Albert Infirmary, Winsford; Alexandra Hospital for Children, Queen's Square; Andover Cottage Hospital; Anti-Vivisection Hospital; Ashford Cottage Hospital. Bath Royal United Hospital; Beckett Hospital; Belgrave Hospital for Children; Birkenhead Borough Hospital; Birkenhead Maternity Hospital; Birmingham Dental Hospital; Birmingham General Hospital; Birmingham Royal Orthopædic Hospital; Blackheath and Charlton Cottage Hospital; Blandford Cottage Hospital; Bootle Borough Hospital; Bushey Heath Cottage Hospital; Burton-on-Trent General Infirmary. Cameron Hospital; Capel Village Hospital; Cardiff Infirmary; Caterham Cottage Hospital; Central London Throat and Ear Hospital; Charing Cross Hospital; Chelmsford and Essex Hospital; Cheshunt Cottage Hospital; Chelsea Hospital for Women; Chesterfield and North Derbyshire Hospital; Chichester Infirmary; City of London Lying-in Hospital; Clapham Maternity Hospital; Coventry and Warwickshire Hospital; Cranleigh Village Hospital; Croydon General Hospital. Darlington Hospital; David Lewis Northern Hospital, Liverpool; Deal and Walmer Victoria Hospital; Devonport Royal Albert Hospital; Dewsbury and District General Hospital; Dumfries and Galloway Royal Infirmary. East London Children's Hospital; East Suffolk and Ipswich Hospital; Edinburgh Royal Infirmary; Egham Cottage Hospital; Enfield Cottage Hospital; Essex County Hospital, Colchester; Evelina Hospital; Eversfield Chest Hospital; Evesham Cottage Hospital. Faringdon Cottage Hospital; Friedenheim Hospital; Frimley District Hospital. Glasgow Eye Infirmary; Glasgow Maternity and Women's Hospital; Glasgow Royal Infirmary; Glasgow Royal Samaritan Hospital; Glasgow Victoria Infirmary; Glasgow Western Infirmary; Gloucestershire Royal Infirmary; Gravesend Hospital; Great Northern Central Hospital; Great Western Railway Accident Hospital. Hawick Cottage Hospital; Haydock Cottage Hospital; Home for Mothers and Babies, Woolwich; Hospital for Epilepsy and Paralysis; Hospital for Sick Children, Great Ormond Street; Huntingdon County Hospital. Jessop Hospital for Women, Sheffield. Kensington and Fulham General Hospital; Kensington Dispensary and Children's Hospital; Kettering and District General Hospital; King Edward VII. Hospital, Windsor. Lady Forrester's Hospitals; Lady Margaret Hospital; Leeds General Infirmary; Leicester Infirmary; Leith Hospital; Lincoln County Hospital; Liverpool Dental Hospital; Liverpool Mill Road Infirmary; Liverpool Royal Southern Hospital; Liverpool Stanley Hospital; London Hospital; London Homœpathic Hospital; London Lock Hospital; London Skin Hospital. Manchester Children's Hospital; Manchester Jewish Hospital; Margate Cottage Hospital; Maryport Cottage Hospital; Metropolitan Hospital; Middlesex Hospital; Mildmay Hospitals; Miller General Hospital; Mount Vernon Hospital; Montgomery County Hospital; Morecambe Queen Victoria Cottage Hospital. National Hospital for Diseases of the Heart; New Hospital for Women; Newbury District Hospital; Newcastle Royal Victoria Infirmary; Norfolk and Norwich Hospital; Northampton General Hospital; North Staffordshire Infirmary. Poplar and Stepney Sick Asylum; Preston Hospital; Prince of Wales' General Hospital. Queen Charlotte's Hospital. Radcliffe Infirmary, Oxford; Ramsgate General Hospital; Richmond Royal Hospital; Rochdale Infirmary and Dispensary; Romford Victoria Cottage Hospital; Royal Bucks Hospital; Royal Devon and Exeter Hospital; Royal Free Hospital; Royal Hospital for Diseases of the Chest; Royal Lancaster Infirmary; Royal Mineral Water Hospital, Bath; Royal National Orthopædic Hospital; Royal Surrey County Hospital; Royal Waterloo Hospital for Children and Women; Royal Westminster Ophthalmic Hospital. St. Alban's and Mid-Herts Hospital; St. Bartholomew's Hospital; St. Bartholomew's Hospital, Rochester; St. George's Hospital; St. Helen's Hospital; St. Helen's Providence Free Hospital; St. John's Hospital; St. John's Hospital, Twickenham; St. Mary's Hospital, Plaistow; St. Marylebone Dispensary for Prevention of Consumption; St. Monica's Home; St. Paul's Hospital; St. Peter's Hospital; St. Thomas's Hospital; Seamen's Hospital; Seaside Convalescent Home; South Devon and East Cornwall Hospital; Southport Infirmary; Southend Victoria Hospital; Stamford Street Skin Hospital; Stratton Cottage Hospital; Sunderland Royal Infirmary; Surbiton Cottage Hospital; Swansea General and Eye Infirmary. Tunbridge Wells General Hospital; Tunbridge Wells Homœopathic Hospital. Victoria Hospital, Belfast. Wakefield Clayton Hospital; Warrington Hospital; Wellingborough Cottage Hospital; West End Hospital; Western Ophthalmic Hospital; West Bromwich Hospital; West Herts Hospital; West Kent General Hospital; Westminster Hospital; Wolverhampton and District Hospital for Women; Woolwich Cottage Hospital; Worcester General Infirmary; Worthing Hospital. Yeovil and District Hospital.

